# Frailty, HIV Infection, and Mortality in an Aging Cohort of Injection Drug Users

**DOI:** 10.1371/journal.pone.0054910

**Published:** 2013-01-31

**Authors:** Damani A. Piggott, Abimereki D. Muzaale, Shruti H. Mehta, Todd T. Brown, Kushang V. Patel, Sean X. Leng, Gregory D. Kirk

**Affiliations:** 1 Johns Hopkins University School of Medicine, Baltimore, Maryland, United States of America; 2 Johns Hopkins Bloomberg School of Public Health, Baltimore, Maryland, United States of America; 3 University of Washington School of Medicine, Seattle, Washington, United States of America; Rush University, United States of America

## Abstract

**Background:**

Frailty is associated with morbidity and premature mortality among elderly HIV-uninfected adults, but the determinants and consequences of frailty in HIV-infected populations remain unclear. We evaluated the correlates of frailty, and the impact of frailty on mortality in a cohort of aging injection drug users (IDUs).

**Methods:**

Frailty was assessed using standard criteria among HIV-infected and uninfected IDUs in 6-month intervals from 2005 to 2008. Generalized linear mixed-model analyses assessed correlates of frailty. Cox proportional hazards models estimated risk for all-cause mortality.

**Results:**

Of 1230 participants at baseline, the median age was 48 years and 29% were HIV-infected; the frailty prevalence was 12.3%. In multivariable analysis of 3,365 frailty measures, HIV-infected IDUs had an increased likelihood of frailty (OR, 1.66; 95% CI, 1.24–2.21) compared to HIV-uninfected IDUs; the association was strongest (OR, 2.37; 95% CI, 1.62–3.48) among HIV-infected IDUs with advanced HIV disease (CD4<350 cells/mm3 and detectable HIV RNA). No significant association was seen with less advanced disease. Sociodemographic factors, comorbidity, depressive symptoms, and prescription drug abuse were also independently associated with frailty. Mortality risk was increased with frailty alone (HR 2.63, 95% CI, 1.23–5.66), HIV infection alone (HR 3.29, 95% CI, 1.85–5.88), and being both HIV-infected and frail (HR, 7.06; 95%CI 3.49–14.3).

**Conclusion:**

Frailty was strongly associated with advanced HIV disease, but IDUs with well-controlled HIV had a similar prevalence to HIV-uninfected IDUs. Frailty was independently associated with mortality, with a marked increase in mortality risk for IDUs with both frailty and HIV infection.

## Introduction

Frailty is a clinical syndrome which increases in prevalence with age and identifies older persons at higher risk for falls, disability, institutionalization, and death [1], [2]. Conceptualized as a state of diminished reserves due to deficits across multiple physiologic systems, frailty leads to an increased vulnerability and limited adaptability to internal and external stressors [1], [2]. A frailty phenotype, operationalized by Fried and colleagues, predicts adverse clinical outcomes in geriatric populations [3], [4].

Since the advent of highly active antiretroviral therapy (HAART), improved survival of HIV-infected individuals has led to an increasing prevalence of older persons living with HIV [5], [6]. However, several studies suggest that even with guideline concordant care, HIV-infected persons have reduced life expectancy relative to the general population and to HIV-uninfected controls with similar behavioral risk [7], [8].

An estimated 3.4 million persons in the U.S. report injecting drugs at some time in their lifetime and this population of injection drug users (IDUs) has also been aging [9]. IDUs have decreased survival attributable to HIV and to other behaviorally-associated comorbid disease [10], [11], [12].

Limited data exist regarding the determinants of frailty among HIV-infected and drug using populations. A higher prevalence of a modified frailty-related phenotype was observed for HIV-infected men who have sex with men (MSM) compared to HIV-uninfected MSM [13]. An increased prevalence of frailty was also seen among HIV-infected women with limited immunological recovery [14], while in an urban HIV clinic setting, frailty was associated with prior opportunistic infections [15]. To date, no studies have examined frailty in an IDU population. Moreover, while frailty increases mortality risk in older HIV-uninfected persons, the effect of frailty on mortality among HIV-infected and at risk IDUs is unknown.

Ensuring the healthy aging of HIV-infected and at-risk persons may be facilitated by earlier interventions among persons at greatest risk for adverse age-associated clinical outcomes. In the current study, we postulated that frailty may be an appropriate phenotype to identify this high-risk subset. Incorporating the objective criteria originally proposed by Fried, we sought to characterize the demographic, behavioral, and clinical correlates of frailty in an aging cohort of HIV-infected and HIV-uninfected IDUs, and to assess the impact of frailty on mortality in this population.

## Methods

### Study Participants

The AIDS Linked to the IntraVenous Experience (ALIVE) cohort has prospectively followed persons with a history of injecting drugs in a community-recruited cohort since 1988. IDUs aged 18 years or older were recruited through street-based efforts from 1988 through 2008 as previously detailed [16], [17]. The ALIVE study has been continually approved by the Johns Hopkins Institutional Review Board, and all participants provided written informed consent.

### Data Collection

At semi-annual visits, ALIVE participants completed standardized questionnaires and underwent clinical examination. Detailed information obtained at each follow-up visit included socioeconomic, behavioral, and clinical parameters for the prior 6 month period. Substance use including alcohol, tobacco and illicit injection and non-injection drug use were assessed by participant self report of behaviors in the prior 6 month period. Comorbid conditions ascertained included obesity (defined as a body mass index [BMI] ≥30) and participant self-report of any provider diagnosis of diabetes, hypertension, or cerebrovascular, cardiovascular, renal, chronic lung, malignant, or liver disease. Hazardous alcohol use was assessed using the Alcohol Use Disorders Identification Test (AUDIT) [18]. Depressive symptoms were assessed using the Center for Epidemiological Studies Depression Scale (CES-D) [19]. Prescription drug abuse was by participant self report of abuse of drugs prescribed to them by a physician in the last year [20]. HAART was defined as use of at least 3 antiretroviral drugs, 1 of which was a nonnucleoside reverse-transcriptase inhibitor, tenofovir, abacavir, or a protease inhibitor and was reflective of use in the prior 6 months [21].

At each visit, HIV-uninfected persons had antibodies to HIV-1 assayed by enzyme-linked immunosorbent assay, with Western blot confirmation. CD4 cell counts were measured on HIV-infected persons at each visit using flow cytometry, and plasma HIV-1 RNA levels determined using reverse-transcriptase PCR methods. Mortality was assessed through linkage to the National Death Index (NDI) with review of death certificates to confirm correct matches.

### Frailty Assessment

Frailty was assessed using the 5 original Fried criteria: slow gait, decreased grip strength (weakness), poor endurance (exhaustion), low physical activity, and physical shrinking (weight loss) (Table S1) [3]. Frailty assessment was routinely performed in ALIVE at six month intervals from July 2005 through the study period (91% of person-visits assessed), except that in March 2007 the assessment interval was altered to an annual basis until funding was secured to allow semi-annual assessment again in January 2008. For the physical activity domain, in lieu of the Minnesota Activity assessment of kilocalorie expenditure utilized by Fried, we incorporated the self-reported response to a standardized question on physical limitations as previously characterized in the Multicenter AIDS Cohort Study (MACS) [13], [22]. Physical shrinking at each visit was defined as measured weight loss of ≥5% body weight from the prior study visit. For analysis, we included weight assessments that were 5 to 12 months from the last measurement (95% of all measurements). Each frailty parameter was considered as a binary variable (0, 1) and summed to obtain a frailty score; ≥3 was considered frail, 1 or 2 considered prefrail, and scores of 0 considered robust.

### Statistical Analysis

We compared participant characteristics by HIV status and by frailty status at baseline and by person-visits. For each person-visit, frailty was treated as a 3 category outcome (robust, prefrail, frail). Using all person-visits, generalized linear mixed models estimated associations of sociodemographic, behavioral, and clinical factors with the frailty phenotype, comparing frail to robust and prefrail to robust. To account for the intra-person correlation within the repeated frailty measures, participants were incorporated as random effects, with other covariates considered fixed effects [23]. Age was included as a continuous variable. Given the predominance of African Americans in the cohort, self-reported race was dichotomized as African American versus other. Depressive symptomatology was assessed using a modified version of the CES-D scale, removing the 2 items included in the frailty assessment and adjusting the score to consider ≥21 as indicative of depressive symptoms. In sensitivity analyses using variable CES-D cutpoints or including the frailty-associated items, findings were not significantly changed. An AUDIT score of ≥8 was considered to be indicative of hazardous alcohol use [18]. In sensitivity analyses excluding the period of annual frailty assessment, no substantive changes to the covariate associations with prefrailty and frailty were observed.

To evaluate the relationship between prefrailty and frailty with all-cause mortality, Kaplan-Meier survival analyses and Cox proportional hazards regression models were performed. The index (baseline) visit, defined as the first visit for which frailty was measured, was the time origin with observation until date of death or for those remaining alive, December 31, 2008. Frailty status, CD4 count, and HIV viral load were considered as time-varying covariates. To evaluate the independent and joint effects of HIV and frailty on mortality, we constructed a 4-category variable combining HIV status (positive/negative) with frailty status (frail if score ≥3; nonfrail if score 0–2). Given the lack of association of prefrailty (frailty score 1–2) with increased risk of mortality relative to the robust group, we combined the robust group with the prefrail group to create the “nonfrail” group for this 4-category variable. Unadjusted hazard ratios were estimated, with multivariable models constructed based on inclusion of variables found to be associated with the outcome and of variables considered *a priori* to be important predictors of mortality. The proportional hazards assumption was found to be reasonable by graphical assessment. Analyses were performed using STATA (version 11; Stata Corp., College Station, TX).

## Results

A total of 1230 ALIVE participants contributed 3365 person-visits (median of 3 frailty measurements; IQR, 2–4). At initial frailty assessment, the median age of participants was 48 years (IQR, 42.9, 52.5), 89% were African American, 66% were male and 29% were HIV-infected. Of the 3365 person-visits (Table 1), 31% were among HIV-infected persons, with a median CD4 cell count of 296 (IQR, 168, 475) cells/uL, and a median viral load of 2.7 (IQR, 1.6, 4.4) log_10_ copies/ml. Recent HAART use was reported at 54% of visits.

**Table 1:** Characteristics of 1230 ALIVE Participants at 3365 Study Visits, by HIV Status^a^.

	HIV-uninfected	HIV-infected
	N = 2306 visits	N = 1059 visits
	No. (%)	No. (%)
Age, median (IQR), y	49.3 (44.2, 54.0)	48.7 (44.6, 52.8)
Female	751 (32.6)	388 (36.6)
African American	2074 (89.9)	1017 (96.0)
Less than high school education	1318 (57.2)	682 (64.9)
Not married/common law	2109 (91.5)	1004 (95.4)
Homeless^b^	280 (12.2)	112 (10.6)
Hazardous alcohol use^b^	530 (23.0)	196 (18.5)
Recent injection drug use^b^	925 (40.1)	331 (31.3)
Any non-injection drug use^b^	1055 (45.8)	345 (32.6)
Recent tobacco use^b^	1894 (82.2)	851 (80.7)
Prescription drug abuse^c^	244 (10.6)	58 (5.5)
Depressive symptoms^b^	491 (21.3)	212 (20.0)
# Comorbid Conditions^d^		
0–1	1636 (72.2)	770 (73.8)
2	373 (16.5)	173 (16.6)
≥3	257 (11.3)	101 (9.7)
CD4^+^ cell count, median (IQR)		296 (168, 475)
HIV RNA, median (IQR), log_10_ copies/ml		2.66 (1.60, 4.43)
Median CD4^+^ nadir (IQR)		135 (53, 230)
Recent HAART^b^		572 (54.2)

Abbreviations: HAART, highly active antiretroviral therapy; IQR, interquartile range; y, years; Hazardous alcohol use, score of ≥8 on the AUDIT; Depressive symptoms, score of ≥21 on the CES-D.

a Data are no. (%) of participants, unless otherwise indicated.

b Reflect characteristics within the previous 6 months.

c Reflect characteristics within the prior year.

d Diabetes, Hypertension, Cerebrovascular accident, Cardiovascular disease, Renal disease, Chronic obstructive pulmonary disease, Cancer, Obesity, Liver disease.

At the index visit, 12.3% of participants were classified as frail and 62.1% as prefrail. Among all 3365 person-visits, 12.4% (417 person-visits) met criteria for frailty and 60% (2020 person-visits) criteria for prefrailty. Univariate and multivariate associations with frailty and prefrailty are shown in Table 2. In multivariable analysis, frailty was significantly associated with older age, female gender, lower educational attainment, absence of a cohabitating partner, depressive symptoms, and increased number of comorbid conditions (Table 2). In univariate analysis, frailty was associated with hazardous alcohol use, being homeless, and non-injection use of illicit drugs. However, these associations did not retain significance in multivariable analyses. HIV infection was associated with a 66% greater likelihood of frailty (OR, 1.66; 95% CI, 1.24–2.21) (Table 2, Model A). In further analysis of HIV disease status (Table 2, Model B), having both immunosuppression (CD4 count <350 cells) and a detectable viral load was significantly associated with frailty (OR, 2.37; 95% CI, 1.62–3.48). There was no significant difference in frailty between HIV-uninfected person-visits and those of HIV-infected persons with a CD4 count ≥350 cells and an undetectable viral load while modest, but non-significant associations were seen for HIV-infected IDUs with either lower CD4 counts only or detectable viral load only. In joint analysis of current and nadir CD4 count, current CD4 count but not CD4 nadir was found to be significantly associated with frailty (data not shown). In separate adjusted models, HIV-infected IDUs not receiving HAART had a substantially greater likelihood of frailty (OR, 1.91; 95% CI, 1.32–2.75) compared to HIV-uninfected IDUs, although this association was substantially attenuated but remained significant with recent HAART usage (OR, 1.45; 95% CI, 1.01–2.07) (Table 2, Model C). In models stratifying by severity of HIV disease, frailty was consistently associated with advanced (but not less advanced) HIV disease irrespective of HAART use.

**Table 2:** Factors Associated with Frailty and Prefrailty among 3365 ALIVE Study Person-Visits^a^.

Model A. Odds of Frailty and Prefrailty by Sociodemographic, Behavioral, and Clinical Risk Factors^b^					
	Prefrail	Prefrail	Frail		Frail
	Unadjusted	Adjusted	Unadjusted		Adjusted
	OR (95% CI)	OR (95% CI)	OR (95% CI)		OR (95% CI)
Age (per year)	1.00 (0.99, 1.01)	1.02 (1.00, 1.03)	1.03 (1.01, 1.05)		1.05 (1.03, 1.07)
Female	1.24 (1.02, 1.51)	1.17 (0.95, 1.45)	1.62 (1.23, 2.13)		1.44 (1.07, 1.94)
African American	0.72 (0.52, 1.00)	0.72 (0.50, 1.04)	0.76 (0.47, 1.23)		0.67 (0.40, 1.13)
Less than high school education	1.27 (1.05, 1.54)	1.25 (1.03, 1.52)	1.43 (1.09, 1.87)		1.48 (1.12, 1.95)
Not married/common law	1.56 (1.08, 2.23)	1.57 (1.08, 2.29)	1.77 (1.02, 3.06)		2.05 (1.16, 3.60)
Homeless^d^	1.23 (0.93, 1.61)	–	1.49 (1.06, 2.11)		–
Hazardous alcohol use^d^	1.20 (0.98, 1.48)	–	1.47 (1.12, 1.92)		–
Recent injection drug use^d^	1.04 (0.87, 1.23)	–	1.00 (0.79, 1.27)		–
Any non-injection drug use^d^	1.21 (1.01, 1.44)	–	1.38 (1.09, 1.75)		–
Recent tobacco use^d^	1.11 (0.88, 1.41)	–	1.32 (0.94, 1.85)		–
Prescription drug abuse^e^	1.66 (1.20, 2.29)	1.50 (1.05, 2.14)	2.14 (1.45, 3.14)		1.70 (1.11, 2.59)
Depressive symptoms^d^	2.23 (1.77, 2.81)	2.11 (1.66, 2.69)	4.70 (3.58, 6.16)		4.40 (3.31, 5.84)
# Comorbid conditions^f^					
0–1	Ref	Ref	Ref		Ref
2	1.08 (0.85, 1.38)	1.05 (0.82, 1.35)	1.87 (1.40, 2.49)		1.70 (1.26, 2.30)
≥3	1.18 (0.87, 1.60)	1.10 (0.80, 1.51)	2.67 (1.84, 3.87)		2.06 (1.39, 3.05)
HIV negative	Ref	Ref	Ref		Ref
HIV positive	1.34 (1.09, 1.64)	1.38 (1.11, 1.70)	1.53 (1.15, 2.02)		1.66 (1.24, 2.21)
**Model B. Odds of Frailty and Prefrailty by CD4 and VL Strata** c					
	**Prefrail**	**Prefrail**	**Frail**	**Frail**	
	**Unadjusted**	**Adjusted**	**Unadjusted**	**Adjusted**	
	**OR (95% CI)**	**OR (95% CI)**	**OR (95% CI)**	**OR (95% CI)**	
HIV negative	Ref	Ref	Ref	Ref	
CD4≥350, VL UD	1.10 (0.79, 1.55)	1.18 (0.82, 1.69)	1.03 (0.64, 1.65)	1.09 (0.67, 1.77)	
CD4<350, VL UD	1.23 (0.82, 1.84)	1.33 (0.89, 2.00)	1.35 (0.81, 2.25)	1.47 (0.86, 2.51)	
CD4≥350, VL+	1.06 (0.71, 1.58)	1.04 (0.69, 1.56)	1.33 (0.80, 2.22)	1.37 (0.80, 2.36)	
CD4<350, VL+	1.76 (1.33, 2.33)	1.79 (1.33, 2.39)	2.12 (1.46, 3.07)	2.37 (1.62, 3.48)	
**Model C. Odds of Frailty and Prefrailty by HAART status** c					
	**Prefrail**	**Prefrail**	**Frail**	**Frail**	
	**Unadjusted**	**Adjusted**	**Unadjusted**	**Adjusted**	
	**OR (95% CI)**	**OR (95% CI)**	**OR (95% CI)**	**OR (95% CI)**	
HIV negative	Ref	Ref	Ref	Ref	
HAART+	1.30 (1.01, 1.67)	1.40 (1.08, 1.81)	1.27 (0.90, 1.80)	1.45 (1.01, 2.07)	
No HAART^d^	1.38 (1.07, 1.80)	1.35 (1.03, 1.77)	1.87 (1.31, 2.65)	1.91 (1.32, 2.75)	

Abbreviations : HAART, highly active antiretroviral therapy; VL, HIV viral load; UD, undetectable, <50 HIV RNA copies/ml; Hazardous alcohol use, score of ≥8 on the AUDIT; Depressive symptoms, score of ≥21 on the CES-D.

– Not included in final model/not significant in adjusted analyses.

a Data are given as unadjusted and adjusted odds ratios (95% confidence interval).

b Adjusted for age, gender, race, education, marital status, prescription drug abuse, depressive symptoms, # comorbid conditions and HIV status.

c Adjusted for age, gender, race, education, marital status, prescription drug abuse, depressive symptoms, and # comorbid conditions.

d Reflect characteristics within the previous 6 months.

e Reflect characteristics within the prior year.

f Diabetes, Hypertension, Cerebrovascular accident, Cardiovascular disease, Renal disease, Chronic obstructive pulmonary disease, Cancer, Obesity, Liver disease.

In adjusted analysis of prefrailty, factors significantly associated with frailty generally remained similarly associated, but the associations were more modest in magnitude (Table 2), with the exception that comorbidity was not associated with prefrailty. HIV-infected IDUs had a 38% greater likelihood of prefrailty compared to HIV-uninfected IDUs (OR, 1.38; 95% CI, 1.11–1.70), and having a CD4 count<350 and detectable HIV viral load was significantly associated with prefrailty (OR, 1.79; 95% CI, 1.33–2.39).

**Figure 1:**
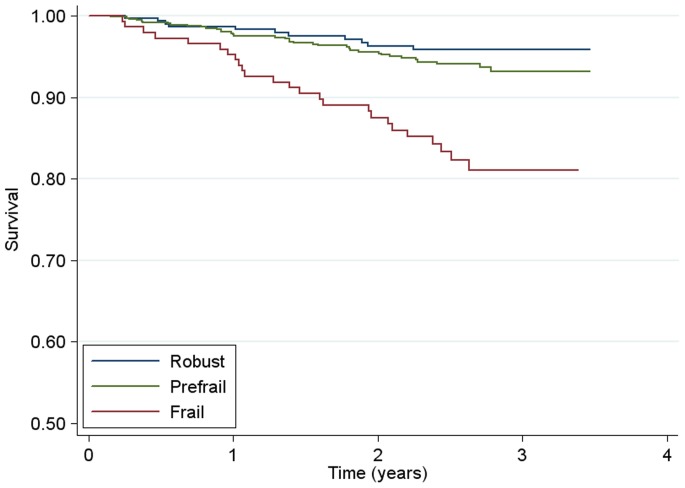
Survival by Frailty Status in the ALIVE cohort. Kaplan Meier Survival Curve Estimates for 1230 ALIVE Participants from July 2005 to December 2008. Robust participants had a frailty score of 0; prefrail participants had a frailty score of 1–2; frail participants had a frailty score of 3–5.

**Figure 2:**
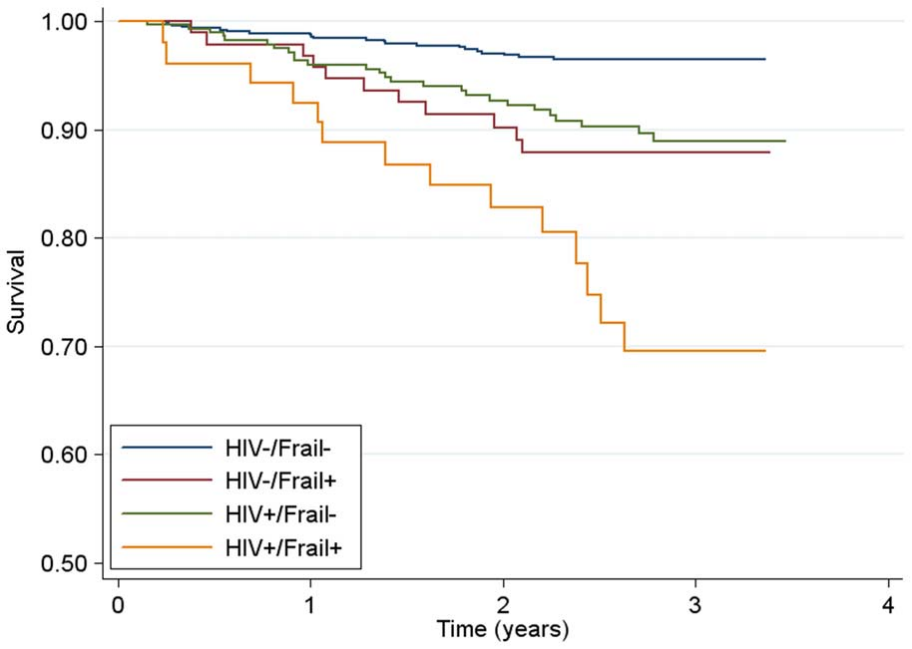
Survival by Frailty and HIV Status in the ALIVE cohort. Kaplan Meier Survival Curve Estimates for 1230 ALIVE Participants from July 2005 to December 2008. Frail- participants had a frailty score of 0–2; Frail+ participants had a frailty score of 3–5.

During prospective evaluation of the relationship of frailty and prefrailty with mortality, we observed 73 deaths over 2644 person-years for a mortality rate of 2.8 per 100 person-years. Overall, frail persons had substantially higher mortality compared to persons that were either prefrail or robust (Figure 1). Adjusting for sociodemographic factors in Cox proportional hazards models, frailty (HR, 2.77; 95% CI, 1.32–5.81), having 3 or more comorbid conditions (HR, 2.97; 95%CI, 1.65–5.35) and HIV infection (HR, 3.05; 95%CI, 1.89–4.93) were independently associated with mortality (Table 3, Model A). Controlling for frailty status and comorbidity, HIV-infected IDUs with advanced disease had notably increased mortality risk (HR, 5.83; 95% CI, 3.48–9.74), with no significantly increased risk of death for those with less advanced HIV disease compared to HIV negatives (Table 3, Model B). The prefrail state was not a significant predictor of death in these models. In models with HIV-uninfected, nonfrail persons as the referent group (Figure 2; Table 3, Model C) being HIV-infected or being frail conferred an increased mortality risk with an approximately 3-fold magnitude for each. Persons that were both HIV-infected and frail had an over 7-fold increased risk of death (HR, 7.06; 95% CI, 3.49–14.3).

**Table 3:** Mortality Risk associated with Frailty and HIV among ALIVE Participants^a^.

	Unadjusted	Adjusted
	HR (95% CI)	HR (95% CI)
**Model A** b		
Age (per year)	1.05 (1.01, 1.08)	1.04 (1.00, 1.08)
Female	1.55 (0.98, 2.46)	1.23 (0.74, 2.02)
African American	1.03 (0.45, 2.38)	0.76 (0.33, 1.77)
Less than high school education	0.86 (0.54, 1.37)	0.79 (0.49, 1.27)
# Comorbid conditions		
0–1	Ref	Ref
2	1.77 (0.95, 3.28)	1.39 (0.73, 2.63)
≥3	4.10 (2.39, 7.03)	2.97 (1.65, 5.35)
HIV positive	2.83 (1.78, 4.49)	3.05 (1.89, 4.93)
Frailty status^d^		
Robust	Ref	Ref
Prefrail	1.46 (0.74, 2.86)	1.24 (0.63, 2.45)
Frail	4.38 (2.16, 8.90)	2.77 (1.32, 5.81)
**Model B** c		
HIV status		
HIV negative	Ref	Ref
CD4≥350, VL UD	0.68 (0.16, 2.83)	0.60 (0.14, 2.55)
CD4<350, VL UD	1.05 (0.25, 4.39)	1.18 (0.28, 4.95)
CD4≥350, VL+	1.12 (0.34, 3.65)	1.38 (0.42, 4.54)
CD4<350, VL+	4.89 (3.01, 7.97)	5.83 (3.48, 9.74)
Frailty status^d^		
Robust	Ref	Ref
Prefrail	1.46 (0.74, 2.86)	1.13 (0.57, 2.22)
Frail	4.38 (2.16, 8.90)	2.20 (1.03, 4.68)
**Model C** c		
HIV/Frailty status^d^		
HIV negative/nonfrail	Ref	Ref
HIV negative/frail	3.45 (1.67, 7.12)	2.63 (1.23, 5.66)
HIV positive/nonfrail	2.85 (1.62, 5.04)	3.29 (1.85, 5.88)
HIV positive/frail	8.31 (4.25, 16.3)	7.06 (3.49, 14.3)

a Data are given as unadjusted and adjusted hazard ratios (95% confidence interval).

b Adjusted for age, gender, race, education, # comorbid conditions, HIV status and frailty status.

c Adjusted for age, gender, race, education and # comorbid conditions.

d Robust participants had a frailty score of 0; prefrail participants had a frailty score of 1–2; frail participants had a frailty score of 3–5; nonfrail participants had a frailty score of 0–2.

## Discussion

In this study, we incorporated standardized assessment of frailty into a community cohort of HIV-infected and epidemiologically-comparable HIV-uninfected IDUs. We identified a frailty prevalence of 12.3% and found that HIV infection, particularly advanced disease stage with lower CD4 cell counts and the absence of ART or virological suppression, was strongly associated with frailty. Despite our cohort being relatively young compared to geriatric populations where frailty has been shown to presage adverse clinical outcomes, frailty was independently associated with increased mortality risk in prospective analysis even after accounting for sociodemographic variables, comorbidity, and HIV infection. Moreover, the combined effect of frailty and HIV on mortality appeared to exceed what one would expect from the additive effect of the individual exposures. In summary, these findings suggest that frailty is a useful phenotype for investigating aging among HIV-infected IDUs and could potentially identify individuals at high-risk for adverse outcomes among these highly vulnerable groups. Our data also raise the possibility that optimal HIV care with virological suppression could attenuate the development of frailty.

Aberrant inflammation and immune dysregulation have been hypothesized to characterize the natural process of aging as well as underlie the pathogenesis of chronic HIV disease [24]. Based on emerging epidemiologic, clinical, and mechanistic evidence, inflammation and immune dysfunction are postulated to play a central role in frailty pathophysiology among older HIV-uninfected adults [25], [26], [27]. We find that HIV-infected IDUs with well-controlled HIV disease are no more likely to be frail than similar individuals without HIV infection. In contrast, the likelihood of frailty was significantly higher with advanced HIV disease with inadequate virologic control. These results suggest that HIV infection without effective treatment may represent a significant, modifiable risk factor for frailty. Thus, together with data from other HIV cohorts [13], [14], these findings suggest a putative role for HAART in arresting the progression to frailty. HAART has been shown to have a significant impact on morbidity and mortality for HIV-infected populations in general and HIV-infected IDUs specifically [28], [29], [30], [31], [32]. The negative impact of late initiation of care and premature interruption of HAART on survival has also been well defined [7], [33], [34]. Engagement in care and adherence to antiretroviral regimens continue to be a daunting challenge for the HIV-infected population [35]. These effects are exacerbated among IDUs, who tend to have later diagnosis, poorer access to care, lower HAART uptake, more limited adherence, and frequent treatment interruptions with significant consequences for HIV-related morbidity and mortality [17], [36], [37], [38], [39], [40], [41]. With less successful navigation of the HIV care continuum, HIV-infected IDUs remain at higher risk for HIV/AIDS progression, and by extension may suffer increased progression to frailty with its consequent adverse outcomes. Future investigations will need to elucidate the underlying mechanisms of frailty development in the setting of HIV and determine whether earlier ART may be effective for frailty prevention.

We found that frailty was independently associated with an increased risk of death among HIV-infected and at risk IDUs. These findings are particularly notable given that the median age of this cohort was 48 years. Frailty has been primarily linked to mortality and other adverse outcomes in significantly older cohorts (predominantly 65 years of age and older) [3], [4]. Consistent with our data, a frailty related construct has demonstrated increased mortality risk in younger populations in 2 recent studies [22], [42]. As comprehensive treatment for HIV infection evolves beyond a focus primarily on viral suppression and broadens to consider management of multiple chronic conditions to achieve healthy aging, assessment tools beyond HIV RNA and CD4 counts will be increasingly needed. Frailty may improve risk stratification and inform appropriate clinical management for aging, complex care patients living with HIV. Further, the joint impact of frailty and HIV infection on mortality suggests that efforts to reduce mortality risk through both frailty and HIV targeted interventions may translate into significant survival benefit.

Besides HIV disease markers, we found that depressive symptoms and prescription drug abuse were associated with both frailty and prefrailty. Previously, we have documented self-medication with ‘street’ drugs for symptoms in this population [43]. Although directionality of these associations cannot be determined in the current analysis, it will be important to examine whether frailty interventions alleviate associated pain or other symptoms and lead to improved health-related quality of life.

Consistent with studies of frailty in HIV-uninfected older adults [3], [44], we observed that advancing age, female gender, lower educational attainment, and increased number of comorbid conditions were associated with frailty within this IDU population. The absence of a cohabiting life partner may be a putative measure of social isolation with which frailty has also been previously associated [3]. As with HIV, dysregulated inflammation has been associated with age and socioeconomic status [24], [45], [46], [47]. Further, hormonal influences have been postulated to play an important role in age-associated changes in inflammation among women [48], [49]. Whether hormonally mediated changes in inflammation account for sex-specific differences in frailty prevalence remains to be determined. However, inflammation may be a predominant pathway by which these factors could contribute to progression to a frail state.

Prefrailty is considered to be an intermediate state that presages progression to frailty in geriatric populations [3]. Therefore, the prefrail state may provide a window of opportunity when interventions could mitigate adverse clinical outcomes. We observed consistent, although attenuated, associations of prefrailty with HIV disease and other frailty risk factors; however, prefrailty was not predictive of mortality. A larger study size or longer duration may allow identification of incremental mortality risk with prefrailty. Further follow-up also is needed to define the likelihood of transition from prefrailty to frailty among HIV-infected persons. These data will be vital to inform development of interventions to reverse or slow progression to frailty. The high prevalence of almost two-thirds of our participants with prefrailty is consistent with other studies and provides a large target population for intervention [3], [4].

Our study had several limitations. Debate persists on the optimal criteria for defining frailty [50]. The frailty phenotype employed in this study closely approximated the original Fried criteria, with objective measurement of weight loss, gait speed, and grip strength. Consistent with prior studies, we substituted a self-reported measure of low physical activity [13], [22]. Our weight loss parameter did not discern intentionality; however, similarly constructed frailty constructs had predictive validity roughly equivalent to the original Fried phenotype in elderly HIV-uninfected persons [4], [51]. As we used weight loss of ≥5% since last study visit (median of 6 months), our threshold for meeting this criteria required greater weight loss than the original criteria. Given the observational nature of the study and lack of temporality for HIV-frailty associations, caution is needed regarding inferences of causality. Our cohort is a predominantly African American, urban IDU cohort and as such, our findings may not be fully generalizable to other HIV-infected populations. However, significant relationships between frailty (and a frailty-related phenotype) and advanced HIV disease have been noted in several non-IDU cohorts [13], [14], [15]. Further, we have observed a similar relationship between frailty and non-HIV-related factors in our population as reported from older HIV-uninfected populations [3], [44]. Whether similar biological mechanisms underlie the development of frailty for these different groups needs to be further investigated. Nevertheless, this population does represent those individuals particularly vulnerable to disparities in access to care and key adverse health care outcomes for whom appropriately targeted frailty interventions could have substantial clinical impact.

Improved understanding of frailty in high-risk populations may translate into clinical utility as well as strengthen our scientific understanding of the aging process. Despite improving survival and recent aging trends, HIV-infected and at risk IDUs continue to experience marked socioeconomic challenges with persistent disparities in treatment access, morbidity and mortality outcomes [10], [11], [17], [21], [52]. Frailty assessment may prove useful in identifying those persons at greatest risk for premature death and allow appropriate intervention. Whether HIV infection and frailty share a single common pathway to premature death remains to be determined. However, elucidation of the mechanisms underlying frailty development may provide substantial opportunities for realizing the healthy aging of HIV-infected and drug using populations, with potential additional benefit to the general aging population.

## Supporting Information

Table S1 **Characterization of the Frailty Phenotype in the AIDS Linked to the IntraVenous Experience (ALIVE) Cohort.**
(DOCX)

## References

[1] Fried LP, Ferrucci L, Darer J, Williamson JD, Anderson G (2004) Untangling the concepts of disability, frailty, and comorbidity: implications for improved targeting and care. J Gerontol A Biol Sci Med Sci 59: 255–263. 15031310 10.1093/gerona/59.3.m255

[2] Xue QL (2011) The frailty syndrome: definition and natural history. Clin Geriatr Med 27: 1–15. 21093718 10.1016/j.cger.2010.08.009 PMC3028599

[3] Fried LP, Tangen CM, Walston J, Newman AB, Hirsch C, et al (2001) Frailty in older adults: evidence for a phenotype. J Gerontol A Biol Sci Med Sci 56: M146–156. 11253156 10.1093/gerona/56.3.m146

[4] Bandeen-Roche K, Xue QL, Ferrucci L, Walston J, Guralnik JM, et al (2006) Phenotype of frailty: characterization in the women’s health and aging studies. J Gerontol A Biol Sci Med Sci 61: 262–266. 16567375 10.1093/gerona/61.3.262

[5] High KP, Brennan-Ing M, Clifford DB, Cohen MH, Currier J, et al (2012) HIV and aging: state of knowledge and areas of critical need for research. A report to the NIH Office of AIDS Research by the HIV and Aging Working Group. J Acquir Immune Defic Syndr 60 Suppl 1S1–18. 22688010 10.1097/QAI.0b013e31825a3668 PMC3413877

[6] Mills EJ, Barnighausen T, Negin J (2012) HIV and aging–preparing for the challenges ahead. N Engl J Med 366: 1270–1273. 22475591 10.1056/NEJMp1113643

[7] Losina E, Schackman BR, Sadownik SN, Gebo KA, Walensky RP, et al (2009) Racial and sex disparities in life expectancy losses among HIV-infected persons in the united states: impact of risk behavior, late initiation, and early discontinuation of antiretroviral therapy. Clin Infect Dis 49: 1570–1578. 19845472 10.1086/644772 PMC2783631

[8] Lohse N, Hansen AB, Pedersen G, Kronborg G, Gerstoft J, et al (2007) Survival of persons with and without HIV infection in Denmark, 1995–2005. Ann Intern Med 146: 87–95. 17227932 10.7326/0003-4819-146-2-200701160-00003

[9] Armstrong GL (2007) Injection drug users in the United States, 1979–2002: an aging population. Arch Intern Med 167: 166–173. 17242318 10.1001/archinte.167.2.166

[10] Wolfe D, Carrieri MP, Shepard D (2010) Treatment and care for injecting drug users with HIV infection: a review of barriers and ways forward. Lancet 376: 355–366. 20650513 10.1016/S0140-6736(10)60832-X

[11] Degenhardt L, Hall W, Warner-Smith M (2006) Using cohort studies to estimate mortality among injecting drug users that is not attributable to AIDS. Sex Transm Infect 82 Suppl 3iii56–63. 16735295 10.1136/sti.2005.019273 PMC2576734

[12] Kohli R, Lo Y, Howard AA, Buono D, Floris-Moore M, et al (2005) Mortality in an urban cohort of HIV-infected and at-risk drug users in the era of highly active antiretroviral therapy. Clin Infect Dis 41: 864–872. 16107987 10.1086/432883

[13] Desquilbet L, Jacobson LP, Fried LP, Phair JP, Jamieson BD, et al (2007) HIV-1 infection is associated with an earlier occurrence of a phenotype related to frailty. J Gerontol A Biol Sci Med Sci 62: 1279–1286. 18000149 10.1093/gerona/62.11.1279

[14] Terzian AS, Holman S, Nathwani N, Robison E, Weber K, et al (2009) Factors associated with preclinical disability and frailty among HIV-infected and HIV-uninfected women in the era of cART. J Womens Health (Larchmt) 18: 1965–1974. 20044858 10.1089/jwh.2008.1090 PMC2828186

[15] Onen NF, Agbebi A, Shacham E, Stamm KE, Onen AR, et al (2009) Frailty among HIV-infected persons in an urban outpatient care setting. J Infect 59: 346–352. 19706308 10.1016/j.jinf.2009.08.008

[16] Vlahov D, Anthony JC, Munoz A, Margolick J, Nelson KE, et al (1991) The ALIVE study, a longitudinal study of HIV-1 infection in intravenous drug users: description of methods and characteristics of participants. NIDA Res Monogr 109: 75–100. 1661376

[17] Salter ML, Lau B, Go VF, Mehta SH, Kirk GD (2011) HIV infection, immune suppression, and uncontrolled viremia are associated with increased multimorbidity among aging injection drug users. Clin Infect Dis 53: 1256–1264. 21976463 10.1093/cid/cir673 PMC3214585

[18] Saunders JB, Aasland OG, Babor TF, de la FuenteJR, Grant M (1993) Development of the Alcohol Use Disorders Identification Test (AUDIT): WHO Collaborative Project on Early Detection of Persons with Harmful Alcohol Consumption–II. Addiction 88: 791–804. 8329970 10.1111/j.1360-0443.1993.tb02093.x

[19] Weissman MM, Sholomskas D, Pottenger M, Prusoff BA, Locke BZ (1977) Assessing depressive symptoms in five psychiatric populations: a validation study. Am J Epidemiol 106: 203–214. 900119 10.1093/oxfordjournals.aje.a112455

[20] Skinner HA (1982) The drug abuse screening test. Addict Behav 7: 363–371. 7183189 10.1016/0306-4603(82)90005-3

[21] Mehta SH, Kirk GD, Astemborski J, Galai N, Celentano DD (2010) Temporal trends in highly active antiretroviral therapy initiation among injection drug users in Baltimore, Maryland, 1996–2008. Clin Infect Dis 50: 1664–1671. 20450418 10.1086/652867 PMC2874101

[22] Desquilbet L, Jacobson LP, Fried LP, Phair JP, Jamieson BD, et al.. (2011) A Frailty-Related Phenotype Before HAART Initiation as an Independent Risk Factor for AIDS or Death After HAART Among HIV-Infected Men. J Gerontol A Biol Sci Med Sci. 10.1093/gerona/glr097 PMC3156632 21719610

[23] Rabe-Hesketh S, Skrondal A (2008) Multilevel and Longitudinal Modeling Using Stata. College Station, TX: Stata Press.

[24] Deeks SG (2011) HIV infection, inflammation, immunosenescence, and aging. Annu Rev Med 62: 141–155. 21090961 10.1146/annurev-med-042909-093756 PMC3759035

[25] Yao X, Li H, Leng SX (2011) Inflammation and immune system alterations in frailty. Clin Geriatr Med 27: 79–87. 21093724 10.1016/j.cger.2010.08.002 PMC3011971

[26] Leng SX, Xue QL, Tian J, Walston JD, Fried LP (2007) Inflammation and frailty in older women. J Am Geriatr Soc 55: 864–871. 17537086 10.1111/j.1532-5415.2007.01186.x

[27] Walston J, McBurnie MA, Newman A, Tracy RP, Kop WJ, et al (2002) Frailty and activation of the inflammation and coagulation systems with and without clinical comorbidities: results from the Cardiovascular Health Study. Arch Intern Med 162: 2333–2341. 12418947 10.1001/archinte.162.20.2333

[28] Detels R, Munoz A, McFarlane G, Kingsley LA, Margolick JB, et al (1998) Effectiveness of potent antiretroviral therapy on time to AIDS and death in men with known HIV infection duration. Multicenter AIDS Cohort Study Investigators. JAMA 280: 1497–1503. 9809730 10.1001/jama.280.17.1497

[29] Hammer SM, Squires KE, Hughes MD, Grimes JM, Demeter LM, et al (1997) A controlled trial of two nucleoside analogues plus indinavir in persons with human immunodeficiency virus infection and CD4 cell counts of 200 per cubic millimeter or less. AIDS Clinical Trials Group 320 Study Team. N Engl J Med 337: 725–733. 9287227 10.1056/NEJM199709113371101

[30] Murphy EL, Collier AC, Kalish LA, Assmann SF, Para MF, et al (2001) Highly active antiretroviral therapy decreases mortality and morbidity in patients with advanced HIV disease. Ann Intern Med 135: 17–26. 11434728 10.7326/0003-4819-135-1-200107030-00005

[31] Vlahov D, Galai N, Safaeian M, Galea S, Kirk GD, et al (2005) Effectiveness of highly active antiretroviral therapy among injection drug users with late-stage human immunodeficiency virus infection. Am J Epidemiol 161: 999–1012. 15901620 10.1093/aje/kwi133 PMC4078731

[32] Wood E, Hogg RS, Lima VD, Kerr T, Yip B, et al (2008) Highly active antiretroviral therapy and survival in HIV-infected injection drug users. JAMA 300: 550–554. 18677027 10.1001/jama.300.5.550

[33] Kitahata MM, Gange SJ, Abraham AG, Merriman B, Saag MS, et al (2009) Effect of early versus deferred antiretroviral therapy for HIV on survival. N Engl J Med 360: 1815–1826. 19339714 10.1056/NEJMoa0807252 PMC2854555

[34] Giordano TP, Gifford AL, White AC Jr, Suarez-Almazor ME, Rabeneck L, et al (2007) Retention in care: a challenge to survival with HIV infection. Clin Infect Dis 44: 1493–1499. 17479948 10.1086/516778

[35] Gardner EM, McLees MP, Steiner JF, Del Rio C, Burman WJ (2011) The spectrum of engagement in HIV care and its relevance to test-and-treat strategies for prevention of HIV infection. Clin Infect Dis 52: 793–800. 21367734 10.1093/cid/ciq243 PMC3106261

[36] Wood E, Hogg RS, Harrigan PR, Montaner JS (2005) When to initiate antiretroviral therapy in HIV-1-infected adults: a review for clinicians and patients. Lancet Infect Dis 5: 407–414. 15978527 10.1016/S1473-3099(05)70162-6

[37] Nijhawan A, Kim S, Rich JD (2008) Management of HIV infection in patients with substance use problems. Curr Infect Dis Rep 10: 432–438. 18687208 10.1007/s11908-008-0068-x PMC2936230

[38] Arnsten JH, Demas PA, Grant RW, Gourevitch MN, Farzadegan H, et al (2002) Impact of active drug use on antiretroviral therapy adherence and viral suppression in HIV-infected drug users. J Gen Intern Med 17: 377–381. 12047736 10.1046/j.1525-1497.2002.10644.x PMC1495042

[39] Celentano DD, Vlahov D, Cohn S, Shadle VM, Obasanjo O, et al (1998) Self-reported antiretroviral therapy in injection drug users. JAMA 280: 544–546. 9707145 10.1001/jama.280.6.544

[40] Kavasery R, Galai N, Astemborski J, Lucas GM, Celentano DD, et al (2009) Nonstructured treatment interruptions among injection drug users in Baltimore, MD. J Acquir Immune Defic Syndr 50: 360–366. 19214124 10.1097/QAI.0b013e318198a800 PMC2782439

[41] Mathers BM, Degenhardt L, Ali H, Wiessing L, Hickman M, et al (2010) HIV prevention, treatment, and care services for people who inject drugs: a systematic review of global, regional, and national coverage. Lancet 375: 1014–1028. 20189638 10.1016/S0140-6736(10)60232-2

[42] Rockwood K, Song X, Mitnitski A (2011) Changes in relative fitness and frailty across the adult lifespan: evidence from the Canadian National Population Health Survey. CMAJ 183: E487–494. 21540166 10.1503/cmaj.101271 PMC3091935

[43] Khosla N, Juon HS, Kirk GD, Astemborski J, Mehta SH (2011) Correlates of non-medical prescription drug use among a cohort of injection drug users in Baltimore City. Addict Behav 36: 1282–1287. 21868170 10.1016/j.addbeh.2011.07.046 PMC3179799

[44] Szanton SL, Seplaki CL, Thorpe RJ Jr, Allen JK, Fried LP (2010) Socioeconomic status is associated with frailty: the Women’s Health and Aging Studies. J Epidemiol Community Health 64: 63–67. 19692719 10.1136/jech.2008.078428 PMC2856660

[45] Carroll JE, Cohen S, Marsland AL (2011) Early childhood socioeconomic status is associated with circulating interleukin-6 among mid-life adults. Brain Behav Immun. 10.1016/j.bbi.2011.05.016 PMC3175292 21672624

[46] Nazmi A, Victora CG (2007) Socioeconomic and racial/ethnic differentials of C-reactive protein levels: a systematic review of population-based studies. BMC Public Health 7: 212. 17705867 10.1186/1471-2458-7-212 PMC2018719

[47] Koster A, Bosma H, Penninx BW, Newman AB, Harris TB, et al (2006) Association of inflammatory markers with socioeconomic status. J Gerontol A Biol Sci Med Sci 61: 284–290. 16567379 10.1093/gerona/61.3.284

[48] Joseph C, Kenny AM, Taxel P, Lorenzo JA, Duque G, et al (2005) Role of endocrine-immune dysregulation in osteoporosis, sarcopenia, frailty and fracture risk. Mol Aspects Med 26: 181–201. 15811434 10.1016/j.mam.2005.01.004

[49] Singh T, Newman AB (2011) Inflammatory markers in population studies of aging. Ageing Res Rev 10: 319–329. 21145432 10.1016/j.arr.2010.11.002 PMC3098911

[50] Rodriguez-Manas L, Feart C, Mann G, Vina J, Chatterji S, et al.. (2012) Searching for an Operational Definition of Frailty: A Delphi Method Based Consensus Statement. The Frailty Operative Definition-Consensus Conference Project. J Gerontol A Biol Sci Med Sci. 10.1093/gerona/gls119 PMC3598366 22511289

[51] Ensrud KE, Ewing SK, Taylor BC, Fink HA, Cawthon PM, et al (2008) Comparison of 2 frailty indexes for prediction of falls, disability, fractures, and death in older women. Arch Intern Med 168: 382–389. 18299493 10.1001/archinternmed.2007.113

[52] Crystal S, Akincigil A, Sambamoorthi U, Wenger N, Fleishman JA, et al (2003) The diverse older HIV-positive population: a national profile of economic circumstances, social support, and quality of life. J Acquir Immune Defic Syndr 33 Suppl 2S76–83. 12853856

