# Supplementary material for: Frailty, HIV Infection, and Mortality in an Aging Cohort of Injection Drug Users
Source: PLoS One. 2013 Jan 31;8(1):e54910. doi: 10.1371/journal.pone.0054910 (PMC3561408; doi:10.1371/journal.pone.0054910)
Supplement: Table S1 — Characterization of the Frailty Phenotype in the AIDS Linked to the IntraVenous Experience (ALIVE) Cohort. (DOCX) [file pone.0054910.s001.docx]

| **Table S1: Characterization of the Frailty Phenotype in the AIDS Linked to the IntraVenous Experience (ALIVE) Cohort**   \| **Weight loss** \| Greater than or equal to 5% loss of body weight since prior visit by direct measurement \| \| --- \| --- \| \| **Low physical activity** \| “Does your health now limit the kinds or amounts of vigorous activities you can do, like lifting heavy objects, running, or participating in strenuous sports?”  Positive: limited a lot \| \| **Poor endurance** \| “During the past week, I felt that everything I did was an effort” OR  “During the past week, I could not get going” (taken from the Center for Epidemiologic Studies-Depression Scale)  Positive: moderate or most of time for either \| \| **Decreased grip strength** \| Grip strength was assessed using a Jamar dynamometer (Sammons Preston, Bolingbrook, IL). Grip strength was binned into quintiles by gender and BMI.    Positive: lowest 20^th^ percentile by gender and BMI \| \| **Slow gait** \| Time to walk 4m at usual pace. Gait speed was binned into quintiles by gender and median height.  Positive: lowest 20^th^ percentile by gender and median height \| |
| --- | --- | --- | --- | --- | --- | --- | --- | --- | --- | --- |
